# Degenerative and Inflammatory Osteoproliferations in Lumbar Radiographs in Psoriatic Arthritis Patients

**DOI:** 10.3390/jcm11072009

**Published:** 2022-04-03

**Authors:** Gizem Ayan, Abdurrahman Sadic, Levent Kilic, Umut Kalyoncu

**Affiliations:** 1 Division of Rheumatology, Department Internal Medicine, Hacettepe University Medical Faculty, 06230 Ankara, Turkey; drgizemayan@gmail.com (G.A.); drleventkilic@yahoo.com (L.K.); 2 Department Internal Medicine, Hacettepe University Medical Faculty, 06230 Ankara, Turkey; abdurrahman3545@hotmail.com

**Keywords:** psoriatic arthritis, X-ray, lumbar spine

## Abstract

The aim of this study was to determine the distribution different types of osteoproliferative lesions on the lumbar spine and their relations in patients with psoriatic arthritis (PsA) under biologic-disease-modifying anti-rheumatic drug therapy. T12-S1 corners were evaluated in 182/274 (66.4%) patients with lumbar radiographs. Lesions were determined as osteophyte (grade 0–3), erosion, sclerosis, squaring, corner syndesmophyte, and bridging syndesmophyte. Lesions with no clear distinction were defined as ambiguous. The mean (SD) age of 182 PsA (69.2% female) patients was 47.6 years (12.7), the mean age at diagnosis of PsA was 39.7 years (12.7). Of the patients, 112 (61.5%) met the criteria for mNY. Osteophytes were the most frequently detected lesions (42.3%), with 18.1% of patients having osteophyte grade 2 and above (mostly on L2-L4). Syndesmophytes were present in 24.2% of all patients (mostly on T12-L4), and ambiguous lesions were detected in 13 (4.7%) patients. Changes were observed in five ambiguous lesions in patients with follow-up lumbar radiography, four of them transformed into corner syndesmophytes at follow-up, and one was evaluated as osteophyte grade 2. Approximately one fifth of patients presented with significant degenerative new bone formation, and syndesmophytes were found in one fourth. In approximately 7% of all patients, lesions were ambiguous. The nature of these lesions needs to be evaluated in further imaging studies.

## 1. Introduction

New bone formation (NBF) in psoriatic arthritis (PsA) is important in the pathogenesis of the disease; the spine and peripheral entheseal areas are among the most affected areas [1]. NBF may occur as a result of the inflammatory process, as well as due to degenerative changes. The heel is one of the most commonly affected areas in PsA. Osteoproliferation can be seen both in the Achilles tendon and in the plantar region [2]. Plantar spur formation was reported in three quarters of PsA patients and about half of the control group. While osteoproliferation of the Achilles tendon is present in one third of the patients, it can also be detected in the control group. Some radiographic findings can be used to differentiate inflammatory from degenerative; for example, changes such as the size of the spur (dimension) and the detection of fluffy plantar periostitis may be used in this regard [2]. One of the areas with osteoproliferation in PsA and osteoarthritis (OA) patients is the small joints of the hand. In the high-resolution quantitative computed tomography (QCT) study, although there was no difference in the number and size of bone spurs in PsA and hand OA patients, there was a significant difference in the location and distribution of the lesions. For example, while spur formations are more prominent in the entheseal areas in PsA patients, spur formations are more prominent in the cartilage–bone interphase and joint margin in OA patients [3]. In summary, although osteoproliferative changes are frequently seen in peripheral joints in PsA patients, these lesions can also be seen in patients with degenerative diseases.

The same is valid for the spine and vertebral edges, which can become inflamed in PsA, as a representative of enthesitis at the insertions of the anterior and posterior longitudinal ligaments, resulting in various lesions such as syndesmophytes (particularly non-marginal in PsA), erosions, squaring, and paravertebral ossifications [4,5]. For this reason, one of the target areas in terms of NBF in PsA patients is the spine. Lumbar, thoracic, and cervical regions can be affected separately in the spine [6]. Unlike ankylosing spondylitis (AS), the mean age of patients with spinal involvement in PsA patients is higher than in AS [7]. Due to this age difference, degenerative changes in the spine are more common in PsA patients. In a study, osteoarthritic changes in the spine were reported in 87.5% of axial PsA (AxPsA) patients with a disease duration of 18 years [7]. In conclusion, differentiation of degenerative/inflammatory osteoproliferative lesions in the spine of PsA patients constitutes an important clinical problem. Inflammatory osteoproliferative lesions in the spine are called syndesmophytes (marginal and non-marginal), and degenerative osteoproliferative lesions are called osteophytes [8,9]. Scores such as the modified Stoke Ankylosing Spondylitis Spinal Score (mSASSS), which are frequently used in the evaluation of syndesmophytes, were originally derived from AS patients, and then they were applied in PsA patients [10,11]. In the following period, scores such as the Psoriatic Arthritis Spondylitis Radiology Index (PASRI), which were shown to be better in sensitivity and specificity in PsA, were developed [12]. It has been shown that the PASRI score is superior to both mSASSS and The Bath Ankylosing Spondylitis Radiology Index (BASRI) spine scores in terms of parameters including reliability and sensitivity to change [7,12,13]. One of the important differences of the PASRI score is that anterior–posterior (AP) radiographs are also evaluated in addition to lateral radiographs.

While evaluating scores such as mSASSS and PASRI in patients with AxPsA, degenerative lesions are not emphasized much. In a conventional X-ray, degenerative (osteophyte) and inflammatory (syndesmophyte) NBF differentiation can be easily performed in many patients and vertebral units. However, there is a group of patients whose ambiguous lesions cannot be clearly distinguished from each other by X-ray in daily practice. As far as we know, there is no study in terms of the frequency and distribution of these ambiguous lesions and other factors that may affect these lesions in PsA. Syndesmophytes are both have diagnostic and prognostic value in spondylarthritis patients; therefore, the differential diagnosis from degenerative lesions is vital [14]. In this study, we aimed (i) to evaluate X-rays and determine the distribution of osteophytes, syndesmophytes, and ambiguous osteoproliferative lesions on lumbar conventional lateral/AP X-rays; (ii) to determine related factors in the PsA cohort under biologic-disease-modifying anti-rheumatic drug (bDMARD) treatment.

## 2. Materials and Methods

### 2.1. Patient Selection

Hacettepe University biological database (HUR-BIO), a single-center database of inflammatory arthritis patients using bDMARDs, was established in 2005. Patients with at least one AP and/or Ferguson pelvic X-ray in the HUR-BIO PsA database were included in the study, regardless of the presence of sacroiliitis on the pelvic X-ray. Patients with PsA were diagnosed by the clinician, but also noted whether they met the Classification Criteria for Psoriatic Arthritis (CASPAR) [15]. Patients with lateral and AP lumbar radiographs were included in the study among patients with pelvic X-ray. Patients whose lumbar radiographs were found unsuitable for evaluation were not included in the study.

### 2.2. Data Collection

Gender, age, psoriasis diagnosis age, PsA symptom onset age, and PsA diagnosis age of PsA patients were recorded. Smoking, BMI at the time of initiation of bDMARD, presence of obesity (whether BMI > 30), and comorbidities (The Charlson Comorbidity Index (CCI)) were recorded. Dactylitis (ever, yes/no), enthesitis (Leeds enthesitis index), nail involvement (pitting, onycholysis, hyperkeratotic changes), radiographic sacroiliitis (according to mNY criteria), HLA-B27, RF, and presence of anti-CCP (ever) were noted. Patient reported outcomes (PROs) and C-reactive protein (CRP) levels were assessed both at the baseline the visit when bDMARDs were initiated and at the last visit in the registry.

### 2.3. Ethical Approval

The study was approved by the Ethics Committee of Hacettepe University Faculty of Medicine (No. GO 15/788) and was performed in accordance with the ethical standards laid down in the 1964 Declaration of Helsinki. Informed consent was obtained from all patients included in the study.

### 2.4. Pelvic and Lumbar X-ray Readings and Definitions

Pelvic and Lumbar X-rays were evaluated by an experienced rheumatologist (U.K.). A proportion of 10% of patients recruited were re-read by the same rheumatologist (U.K.) after a month and another rheumatology specialist (L.K.). The intra- and interrater agreements were determined. 

Lumbar radiographs: First, lumbar (lateral and AP) radiographs of the patients during the follow up after PsA diagnosis were evaluated. If there were more than one lumbar (lateral and AP) radiographs, these follow-up radiographs were also evaluated. If there was a change between the lesions identified during the follow up of the patients, then the last lesion was considered. Lumbar radiographs from the lateral T12 lower and S1 upper parts (182 patients, 2184 sites) were evaluated as previously described for mSASSS and lumbar mSASSS were determined [10,11]. Inflammatory lesions are grouped as follows: erosion, sclerosis, squaring, syndesmophyte (marginal and non-marginal), and bridging syndesmophytes. The total lumbar mSASSS score was calculated for each patient [10,11]. Tiny syndesmophytes that vertically arose from the annular attachment to the vertebral body were classified as marginal, and a vertically oriented, thick and chunky syndesmophytes, arising beyond the annular attachment to the vertebral body were classified as non-marginal syndesmophytes [5]. Degenerative lesions were grouped between stages 0 and 3, according to Lane’s definition, as grade 0 = no, grade 1 = mild, grade 2 = moderate, and grade 3 = severe [9]). In addition, lumbar disc space narrowing, if any, was also recorded.

**Figure 1:**
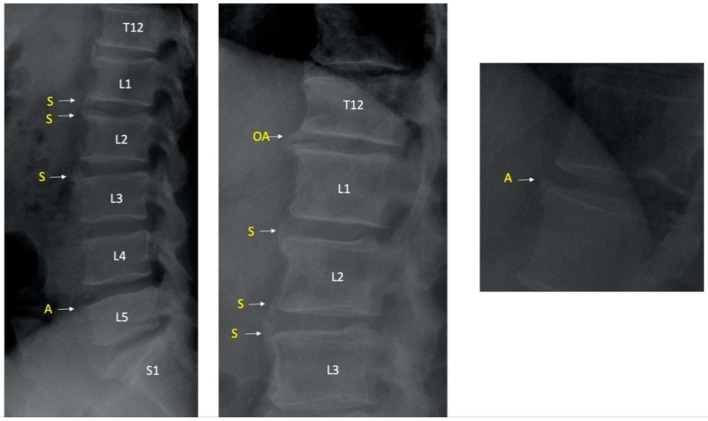
Examples of syndesmophytes and ambiguous lesions. S indicates syndesmophytes and A indicates ambiguous lesions; OA indicates osteophyte. T12 indicates 12th thoracic vertebra and L1/L2/L3/L4/L5 indicate 1st, 2nd, 3rd, 4th, 5th lumbar vertebrae respectively and S1 indicates 1st sacral vertebra.

Lesions that cannot be distinguished from syndesmophytes and osteophytes on lumbar (lateral and AP) radiographs are called ambiguous lesions (Figure 1). 

These lesions were re-read by L.K. after they were determined as ambiguous by U.K., who evaluated all radiographs, and these lesions were grouped as syndesmophytes, osteophytes, and ambiguous lesions by L.K. Lesions with disagreement on the structure were re-evaluated by U.K. and L.K. and a consensus was established.

Lesions between T12 and L5 on AP images of lumbar radiographs were evaluated. The distribution of the lesions was determined using landmarks as follows; S1—upper; L5—lower; L5—upper; L4—lower; L4—upper; L3—lower; L3—upper; L2—lower; L2—upper; L1—lower; L1—upper; T12—lower. These were the case provided that each area was determined separately as right and left. Lumbar AP radiographs were grouped as corner syndesmophyte (marginal or non-marginal), bridging syndesmophyte, osteophyte (grade 1–3), and ambiguous [12].

Pelvic X-ray: Pelvic radiography was also evaluated for sacroiliitis according to mNY criteria (stage 0–4) and new bone formations in the iliac wings (stage 0–4), ischiums (stage 0–4), and symphysis pubis (stage 0–4) were grouped in the pelvis X-ray to give data on the rate of sacroiliitis patients and entheseal changes on the pelvic radiography in patients with or without lesions on the spine [6].

### 2.5. Statistical Analysis

SPSS (version 22.0, IBMVR corp., Armonk, NY, USA) was used to conduct all statistical analyses. Normal distribution was tested visually (histogram, probability plots) and analytically (Kolmogorov–Smirnov skewness and kurtosis). Results were presented as mean (SD) or median (interquartile range (IQR)) for continuous variables and as percentages (frequencies) for categorical variables. According to the distribution status, independent continuous variables were analyzed using Student’s t-test or the Mann–Whitney U test. Dependent continuous variables were analyzed using the paired sample t-test or Wilcoxon test according to the distribution status. Categorical variables were compared using either the chi-square test or Fisher’s exact test where appropriate. The distribution of countable data was expressed as % (percent). Possible factors (age, gender, psoriasis, PsA duration, baseline BMI > 30, CCI, smoking status, PROs, nail involvement, enthesitis, dactylitis, rheumatoid factor, and anti-cyclic citrullinated peptide (Anti-CCP) positivity, HLA-B27 positivity, CRP > 0.8, and presence of sacroiliitis according to mNY criteria) were assessed with univariate analyses and factors with (*p* < 0.20), and were further entered into the logistic regression analysis with backward selection to determine independent predictors of osteophytes > grade 2 and syndesmophytes. Hosmer–Lemeshow goodness-of-fit statistics were used to assess the model fit. A 5% Type-I error level was used to infer statistical significance. The agreement of categorical data was determined by the kappa coefficient (0–0.2—poor; 0.21–0.4—fair; 0.41–0.6—moderate; 0.61–0.8—substantial; 0.81–1—almost perfect) [16].

## 3. Results

### 3.1. Patient Characteristics

Of 362 PsA patients, 274 (75.7%) had at least 1 pelvic X-ray. Lumbar X-ray was present in 182 (66.4%) of 274 patients; 2 lumbar radiographs were found for 53/182 (19.3%) patients, and 3 radiographs were found for 14/182 (5.1%) patients. Patients were prescribed adalimumab (*n* = 75, 42.8%), etanercept (*n* = 31, 17.7%), infliximab (*n* = 31, 17.7%), golimumab (*n* = 10, 5.7%), certolizumab (*n* = 17, 9.7%), secukinumab (*n* = 7, 4%), and ustekinumab (*n* = 4, 2.3%) at the time of lumbar radiography assessment. The demographic and clinical characteristics of 182 patients (69.2% women) with lumbar radiography are given in Table 1, and disease activity parameters at baseline and last visit, after a mean (SD) follow up of 52.8 (47.8) months are given in Table 2.

### 3.2. Intra- and Interrater Agreement

Intrarater agreement showed substantial agreement for the lateral and AP lumbar readings with kappa value 0.728 and 0.631, respectively. Interrater agreement showed moderate agreement for the lateral lumbar readings with the kappa value 0.569 and substantial agreement for AP readings with the kappa value 0.628.

### 3.3. Osteophytes on Lateral Lumbar Radiograph in PsA Patients

Patient level: Osteophytes (≥grade 1) were found in 77 (42.3%) of 182 patients, and 33 (18.1%) patients had ≥grade 2 osteophytes (Table 3). Disc degeneration and narrowing of the lumbar vertebrae were detected in 15/182 (4.3%) of the patients (4 patients L4-L5, 11 patients L5-S1).

Vertebral unit level: Out of a total of 2184 vertebral units, 184 (8.4%) had osteophytes and 75 (3.4%) had ≥ grade 2 osteophytes. Grade 2 and above osteophytes were detected 2.1 times higher in the region upper to the L3 and 2.3 times higher in the upper to the L4.

Factors associated with osteophytes: Those with osteophyte ≥ grade 2 were older [(56.1 (11.8) vs. 45.8 (12.2), *p* < 0.001)], had higher mean (SD) age at diagnosis of PsA [(48.0 (11.2) vs. 37.9 (12.4), <0.001], had baseline BMI >30 (63.6% vs. 37.4%, *p* = 0.006) mean (SD) CCI [2.8 (1.8) vs. 1.8 (1.2), *p* < 0.001], had mean (SD) baseline DAS-28 [4.4 (1.3) vs. 3.6 (1.3), *p* = 0.033)], had mean (SD) last visit DAS-28 [3.1 (1.4) vs. 2.6 (1.2), *p* = 0.022)], and last visit HAQ > 1 was more common (34.4 vs. 17.9%, *p* = 0.038). Multivariate analysis revealed no significant predictors of osteophyte ≥ grade 2 when correction according to the age and gender was made.

### 3.4. Syndesmophytes on Lateral Lumbar Radiograph in PsA Patients

Patient level: At least 1 syndesmophyte was seen in 44 (24.2%) of 182 patients (Table 3). A total of 17/182 (9.3%) patients had bridging syndesmophytes, 36/182 patients (19.8%) had corner syndesmophytes, and 9 patients had both corner and bridging syndesmophytes. Lumbar mSASSS score was 0 in 117/182 (64.3%) patients. Patients with at least 1 point from mSASSS score had a median mSASSS score of 3.0 (IQR 2–6)

Vertebral unit level: Of a total of 2184 vertebral units, 109 (4.9%) had syndesmophytes. While syndesmophytes are more common especially between T12 lower and L2 lower regions, the rate of syndesmophytes has decreased starting from L4 lower vertebra (Table 3). A total of 49/109 (44.9%) syndesmophytes were bridging, while 60 (55.1%) were corner syndesmophytes. A total of 56/60 (93.3%) of the syndesmophytes were marginal and 4 (6.7%) were non-marginal.

Factors associated with syndesmophytes; According to the mNY criteria, sacroiliitis is more common in patients with syndesmophytes (79.5 vs. 55.8%, *p* = 0.005), and the median lumbar mSASSS score was higher [4 (6) vs. 0 (0), *p* < 0.001]. No difference was found in terms of other demographic and clinical characteristics. Multivariate analysis also revealed presence of sacroiliitis [30.4 95% CI (2.1–440.5 *p* = 0.012] and Anti-CCP positivity [0.02 95% CI (0.001–0.415 *p* = 0.013] significantly predicted the syndesmophyte formation.

The median PsA disease duration was similar in patients with bridging and corner syndesmophytes [5.7 (0–5) vs. 8.9 (0–15) years, *p* = 0.084]. Patients with bridging syndesmophytes were more often male than patients with corner syndesmophytes (58.2 vs. 22.2%, *p* = 0.014), and older [mean (SD) age 55.4 (12.1) vs. 46.9 (7.9) years, *p* = 0.010]. Patients with bridging syndesmophytes were more often male (58.2 vs. 28.2%, *p* = 0.008), older [mean (SD) age 55.5 (12.1) vs. 46.9 (12.6) years, *p* = 0.009], and HLA-B27 positivity was more common (60.0 vs. 17.2%, *p* = 0.023).

### 3.5. Ambiguous Lesions on Lateral Lumbar Radiograph in PsA Patients

Patient level: 13 (7.1%) of 182 patients had at least 1 ambiguous lesion (Table 3).

Vertebral unit level: Of the total 2184 vertebral units, 21 (1.0%) had any ambiguous lesion. No difference was found regarding the distribution of ambiguous lesions in the lumbar vertebra (Table 3).

**Figure 2:**
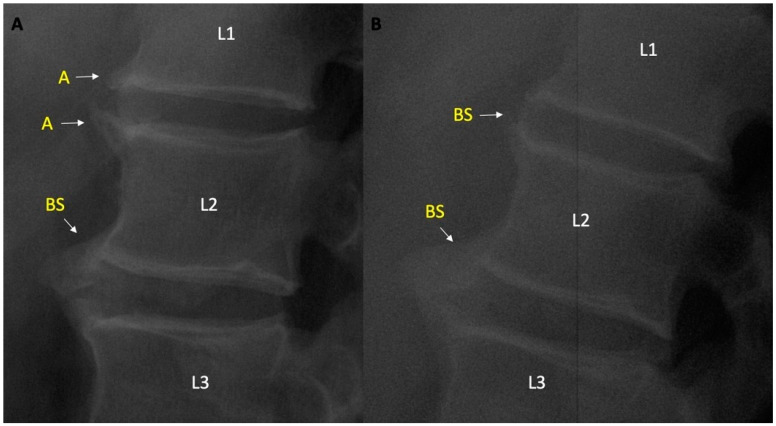
(**A**) Ambiguous lesions on L1 lower and L2 upper vertebral units and BS between L2 lower nad L3 upper vertebral units. (**B**) Turning of Ambiguous lesions to bridging syndesmophytes during follow up. A—ambiguous lesion; BS—bridging syndesmophyte. L1/L2/L3 indicate 1st, 2nd, 3rd lumbar vertebrae respectively.

Ambiguous-lesion-related factors: Patients with ambiguous lesion were older [55.7 (9.8) vs. 47.0 (12.7), *p* = 0.017], had a higher lumbar MSASS score [5.4 (8.1) vs. 1.6 (3.8), *p* = 0.002], corner syndesmophytes (46.1 vs. 17.7%, *p* = 0.013), and bridging syndesmophyte (30.7 vs. 7.7%, *p* = 0.006) were more common, while no difference was found in terms of ≥grade 2 osteophyte (23.1 vs. 17.7%, *p* = 0.63). Changes were observed in 5 ambiguous lesions in patients with follow-up lumbar radiography, after the mean (SD) follow up of 71.2 (24.5) months, 4 of them transformed into syndesmophytes at follow up, and 1 was evaluated as osteophyte grade 2 (Figure 2).

### 3.6. AP Lumbar Radiographs in PsA Patients

Contribution of AP radiography at patient level: when lateral lumbar radiographs are taken into account, 44/182 (24.2%) patients have syndesmophytes. When lateral and AP lumbar radiographs were evaluated together, syndesmophyte was detected in 47/182 (25.8%) patients.

Contribution of AP radiography at syndesmophyte level: There were a total of 109 syndesmophytes on the lateral lumbar radiograph. There were 131 syndesmophytes on lumbar lateral and AP radiographs. Contribution of AP radiographs to the appearance of syndesmophyte was detected in 22/131 (16.8%) vertebral units.

Lumbar AP radiograph showed 60 syndesmophytes, 35 (58.3%) of which were bridging and 25 were corner syndesmophytes. There was complete agreement in 27/60 (45%) syndesmophytes on lumbar AP and lateral radiographs, 22/60 (36.7%) syndesmophytes were not visible on lateral radiographs, whereas they were detected on AP radiographs. A total of 4/60 (6.7%) syndesmophytes were seen as a corner in the lateral radiograph, while it was seen as a bridging in the AP (syndesmophytes progressed when AP was assessed), 7/60 (11.7%) syndesmophyte was seen as a bridging on the lateral, while it was seen as a corner in the AP (syndesmophytes regressed when AP was assessed).

Contribution of AP radiography at ambiguous lesion level: There were ambiguous lesions in 13/182 (7.1%) patients and 21 vertebral units on the lateral lumbar radiograph. There were ambiguous lesions in 5 patients and 6 vertebral units on AP radiographs. When lateral and AP radiographs were examined together, at least 1 ambiguous lesion was detected in 17/182 (9.3%) patients.

## 4. Discussion

In this study, the prevalence and distribution of degenerative (osteophyte) and inflammatory (syndesmophyte) findings of lateral and AP lumbar spine radiographs of PsA patients were evaluated. Degenerative NBFs (≥grade 2 osteophytes) were detected in approximately one fifth of the patients. As expected, patients with degenerative changes were older. On the other hand, osteoproliferation of inflammatory character was found in a quarter of PsA patients. Importantly, approximately 7% of all patients had lesions on the lateral radiographs, and 9% of all patients had lesions on the lateral and AP lumbar radiographs with indistinguishable osteophyte/syndesmophyte lesions.

PsA patients were older than AS patients. Accordingly, it is accepted that the occurrence of degenerative spine changes is more common [7]. On the other hand, there are no detailed studies on the frequency and distribution of osteophytic changes on lateral lumbar radiographs in PsA patients. In this study, when all vertebral units were considered, any osteophyte was found in 8.4% of patients and ≥grade 2 osteophytes were found in 3.4% of patients. The majority of the lesions were mild (grade 1 osteophytes), although moderate–severe osteophytes were also seen in approximately one in five patients. Moderate–severe osteophytic lesions were observed to be more common in the upper part of the vertebral units than in the lower part, especially the upper part of the L4 and L3 vertebrae, where osteophytic lesions are most common. In the Rotterdam prospective cohort, the distribution of grade 2 osteophytes between L1 and L5 was found to be similar in each vertebral area in the lumbar X-ray of approximately 2800 patients aged 55 years and older [17]. It is difficult to make further comments on why osteophytes develop more frequently in some areas in PsA patients, and this requires further confirmation in other studies. In this study, patients with moderate–severe osteophytes were older than the general group, as expected. On the other hand, comorbidity index and especially obesity are seen as an important problem in patients with moderate–severe osteophytes. The Johnston County Osteoarthritis Project has demonstrated the relationship between obesity and spinal osteoarthritis [18]. Considering the relationship between mechanical load and osteoarthritis, this situation should be considered as expected. Disease activity of these patients before bDMARD and during follow-up was higher than patients without osteophytes. This situation can probably be explained by the tendency of the patients to evaluate their general disease activity at a higher rate due to degenerative diseases. There are several treatment modalities used in both psoriasis and PsA starting from basic treatment modalities to more advanced bDMARDs [19,20,21]. On the other hand, functional deterioration generally continues after treatment due to degenerative diseases. As can be understood from all these results, degenerative changes are not uncommonly observed in the lumbar vertebrae of PsA patients, and it should be kept in mind that it may contribute to the activity and functions of the patients, albeit indirectly.

One of the important findings of axial involvement in PsA is sacroiliitis and the other is the development of syndesmophyte. The presence of syndesmophytes in the vertebrae of patients with psoriatic spondylitis, has been reported by Jadon et al. up to 50% [22]. In our study, syndesmophytes were found in one fourth of the lateral lumbar radiographs. If patients with sacroiliitis according to mNY were considered, then syndesmophyte was detected in 31% of patients. In the study by Jadon et al., bridging syndesmophyte was detected 20% among all patients with syndesmophytes, it was found much more frequently in our study as approximately 45% of all syndesmophytes were bridging. This may be related to the fact that our patient group consisted of patients receiving bDMARD treatment and a more severe disease group. There is not much data on who has bridging syndesmophytes in PsA patients. In this study, it was observed that bridging syndesmophytes were more common in male gender, advanced in age, and HLA-B27-positive patients. The relationship of bridging syndesmophytes in AS patients with male gender and advanced age is a well-known phenomenon [23]. From this point of view, our study makes an important contribution to the literature in terms of emphasizing the similarity in the relationship of bridging syndesmophytes in AS and PsA between men and advanced age.

Feld J et al. recently published data on approximately 1300 (37% axial involvement) PsA patients in the Toronto PsA cohort [24]. In this cohort, 19% of patients with axial involvement had HLA-B27 positivity, while 9% of patients with peripheral involvement had a positive HLA-B27 result. In our study, unfortunately, HLA B27 was studied only in one third of the whole group, so it is difficult to make a definitive interpretation. However, in general, 20% of the patients and 27% of the patients who met the mNY criteria have HLA-B27 positivity, while 17% of the patients with corner syndesmophytes and 60% of patients with bridging syndesmophytes have HLA-B27 positivity. This suggests that the relationship between HLA-B27 and severe osteoproliferation may also be present in PsA patients. Recently, it has been shown that HLA B27 is associated with symmetry in syndesmophytes in PsA and AS patients [25]. From the pathogenetic point of view, it has been shown that the development of syndesmophyte formation in AS patients is related to the HLA-B27-mediated tissue-non-specific alkaline phosphatase (TNAP) promoter [26]. On the other hand, Queiro R et al. did not find a relationship between syndesmophyte and HLA-B27 in PsA [27]. While there was no significant difference in patients with corner syndesmophytes in our study, this high HLA-B27 positivity in bridging syndesmophytes needs to be confirmed in a larger patient group. Further studies are needed to explain the pathogenetic mechanism for the relationship with HLA-B27 in PsA patients with bridging syndesmophytes.

It is seen that syndesmophytes occur more frequently especially in the upper part of the lumbar vertebra, in other words above the L2 lower unit. This region is also the area where osteophytic changes can be seen. For this reason, it is an area where it is difficult to distinguish between osteophytes and syndesmophytes from time to time. Not rarely, the same patient has syndesmophyte in the lower part of L2 vertebra, while prominent osteophytes can be seen in the upper part of L3. In cases where the lesions can be clearly distinguished, there is no problem in the evaluation. However, there are cases where it is not possible to distinguish a lesion in a certain vertebral unit from osteophyte and syndesmophyte in a patient group. We named these lesions as ambiguous and at least one of these lesions was detected in 7% of all patients on lateral radiographs and in 9% of lateral and AP radiographs. It has been observed that ambiguous lesions can occur in each vertebral unit, and it is not possible to make a distinction in this respect. On the other hand, in the follow-up lumbar radiographs of the patient with ambiguous lesions in 5 vertebral units, the lesions were evaluated as syndesmophytes in 4 patients, and as stage 2 osteophytes in 1 patient. The fact that the lumbar mSASSS score is significantly higher in patients with an ambiguous lesion in any vertebral unit on the lateral radiograph as corner and bridging syndesmophytes are more frequent. There is no difference in terms of osteophytes, and the lesions evolve into syndesmophytes over time, suggesting that these lesions are more similar to syndesmophytes. It may be appropriate to evaluate the structure of these lesions with more advanced imaging methods, such as low-dose CT, as is performed in peripheral joints [3].

The limitations of this study are that it mainly consisted of patients who were followed up in routine practice. Therefore, some important laboratory tests such as HLA-B27 are missing. Similarly, the lack of follow-up lumbar radiographs in every patient is another limitation. The inadequate follow-up radiographs were insufficient to show how ambiguous lesions develop during follow up. Cervical radiographs were missing in many patients (approximately one third of the patients), because they also included patients in routine practice; therefore, they were not included in the evaluation. It should also be kept in mind that it cannot be generalized to all PsA patients, since the patients consisted of PsA patients using bDMARDs. On the other hand, there are the advantages of this study, such as the detailed evaluation of osteoproliferative lesions in conventional X-ray, evaluation of patients who cannot be differentiated, and the presentation of results of the AP radiographs in addition to lateral radiographs.

## 5. Conclusions

In conclusion, approximately 60% of patients with PsA using bDMARDs have any osteoproliferative lesion on the lumbar radiograph. Osteophytes are the most common lesions and, in one fifth of the patients, ≥grade 2 osteophytes were found. A quarter of the patients had syndesmophytes, while about half of them were bridging syndesmophytes. In this study, male gender, advanced age, and HLA-B27 positivity were related to bridging syndesmophytes in PsA patients for the first time. Even though this result is similar to AS, this needs to be confirmed in other cohorts, while ours represents a patient population requiring bDMARDs. The significant positivity of HLA-B27 in patients with bridging syndesmophytes is a result that should be emphasized, and it needs to be confirmed in larger studies. When lateral and AP lumbar radiographs are evaluated together, approximately 9% of patients have at least one ambiguous lesion. These lesions can probably be interpreted more as syndesmophytes, but further imaging studies and follow-up studies are needed to elucidate the nature of ambiguous lesions.

## Figures and Tables

**Table 1:** Demographic and clinical characteristic of patients according to lateral lumbar radiography findings.

	All Patients *n* = 182	Patients with Normal Lumbar Radiograph *n* = 71	Patients with Any Syndesmophyte *n* = 44	Patients with ≥Grade 2 Osteophyte *n* = 33	Patients with Ambiguous Lesions *n* = 13
Female gender, *n* (%)	126 (69.2)	44 (62.0)	28 (63.6)	25 (75.8)	8 (61.5)
Age at the time of lumbar radiograph, mean (SD), years	44.8 (12.8)	39.6 (12.8)	46.7 (9.9)	52.4 (11.7)	50.9 (8)
PsA diagnosis age, mean (SD), years	39.7 (12.7)	34.8 (12.3)	41.8 (10.7)	48.0 (11.2)	45.5 (9.7)
PsA disease duration until first lumbar radiograph, mean (SD), years	4.8 (6.2)	4.9 (5.4)	4.8 (7.2)	3.2 (7.8)	5 (7.9)
PsO disease duration until first lumbar radiograph, mean (SD), years	13. 7 (11.4)	14.8 (11.5)	13.4 (11.2)	12.4 (13.1)	14.2 (9.6)
PsO/PsA family history, *n* (%)	45 (40.5)	15 (34.9)	14 (50.0)	9 (47.4)	3 (33.3)
PsO start > 40 age, *n* (%)	41 (28.9)	6 (11.1)	9 (25.7)	16 (59.3)	5 (55.6)
BMI, mean (SD) BMI > 30, *n* (%)	29.1 (5.6) 76 (42.2)	27.7 (5.3) 23 (32.2)	29.7 (5.8) 21 (47.7)	30.8 (5.5) 21 (63.6)	32 (4.1) 8 (61.5)
Smoking (ever), *n* (%)	105 (58.3)	46 (65.7)	27 (61.4)	19 (57.6)	8 (61.5)
CCI = 0, *n* (%) CCI = 1, *n* (%) CCI > 1, *n* (%)	90 (51.1) 41 (23.3) 45 (25.5)	45 (64.3) 13 (18.6) 12 (17.0)	19 (46.3) 12 (29.3) 10 (24.3)	8 (25.0) 11 (34.4) 13 (40.0)	3 (23.1) 5 (38.5) 5 (38.5)
RF positivity, *n* (%)	19 (15.0)	7 (14.3)	6 (20.0)	3 (13.0)	2 (18.2)
Anti-CCP positivity, *n* (%)	8 (10.7)	2 (9.5)	4 (20.0)	2 (11.8)	1 (14.3)
HLA-B27 positivity, *n* (%)	13 (20.6)	7 (21.9)	4 (25.0)	1 (12.5)	0 (0)
Dactylitis (ever), *n* (%)	37 (30.6)	16 (34.0)	8 (25.8)	8 (32.0)	5 (50.0)
Enthesitis (ever), *n* (%)	34 (37.0)	14 (36.8)	7 (30.4)	10 (50.0)	2 (28.6)
Nail involvement, *n* (%)	46 (41.4)	22 (48.9)	9 (32.1)	6 (28.6)	1 (11.1)
Sacroiliitis according to mNY criteria, *n* (%)	112 (61.5)	44 (62.0)	35 (79.5)	19 (57.6)	8 (61.5)
Ischium ≥ grade 2, *n* (%)	45 (34.1)	16 (31.4)	14 (31.8)	10 (38.5)	2 (25)
Iliac wing ≥ grade 2, *n* (%)	18 (13.1)	2 (3.6)	8 (18.2)	8 (29.6)	3 (37.5)

PsO—psoriasis; PsA—psoriatic arthritis; BMI—body mass index; CCI—Charlson comorbidity index; RF—rheumatoid factor; CCP—cyclic citrullinated peptide.

**Table 2:** Patient-reported outcomes of patients and CRP levels according to lateral lumbar radiography findings.

		All Patients *n* = 182	Patients with Normal Lumbar Radiograph *n* = 71	Patients with Any Syndesmophyte *n* = 44	Patients with ≥Grade 2 Osteophyte *n* = 33	Patients with Ambiguous Lesions *n* = 13
BASDAI, mean (SD)	Baseline	6.1 (2.1)	6.3 (2.1)	5.8 (1.9)	6.6 (2.1)	7.2 (1.7)
	Last visit	4.4 (2.5)	4.2 (2.7)	4.6 (2.5)	4.9 (2.6)	4.9 (1.8)
BASDAI > 4, *n* (%)	Baseline	92 (83.6)	36 (83.7)	12 (27.2)	20 (95.2)	6 (100)
	Last visit	80 (53)	28 (46.7)	19 (51.4)	16 (61.5)	8 (72.7)
BASFI, mean (SD)	Baseline	4.4 (2.5)	4.3 (2.6)	4.3 (2.8)	5.1 (3.2)	5.9 (2.7)
	Last visit	3.6 (2.5)	3.2 (2.6)	3.6 (2.6)	4.3 (2.6)	3.9 (2.8)
BASFI > 4, *n* (%)	Baseline	56 (53.8)	19 (46.3)	12 (48)	13 (65)	4 (66.7)
	Last visit	59 (42.8)	18 (35.3)	14 (37.8)	15 (55.6)	6 (46.2)
DAS-28, mean (SD)	Baseline	3.7 (1.3)	3.5 (1.3)	3.7 (1.3)	4.4 (1.3)	4.5 (1.3)
	Last visit	2.7 (1.3)	2.5 (1.2)	2.9 (1.5)	3.1 (1.4)	2.9 (1.4)
DAPSA-28, mean (SD)	Baseline	19.2 (9.7)	18.8 (8.6)	17.1 (10.9)	21.2 (13.6)	24.3 (13.9)
	Last visit	11.5 (9.4)	11.5 (8.7)	11.2 (11.9)	13.1 (10.3)	11.8 (9.1)
CRP, mean (SD)	Baseline	1.9 (2.6)	1.5 (1.9)	2.2 (2.8)	1.9 (3.1)	2.9 (3.8)
	Last visit	0.8 (1.4)	1.0 (1.7)	0.7 (1.0)	1.1 (1.9)	0.9 (0.7)
CRP, >0.8, *n* (%)	Baseline	84 (57.1)	30 (51.7)	21 (58.3)	20 (71.4)	9 (81.8)
	Last visit	48 (27.9)	22 (32.4)	9 (22.0)	13 (41.9)	6 (46.2)
HAQ-DI, mean (SD)	Baseline	0.75 (0.58)	0.68 (0.58)	0.73 (0.52)	0.89 (0.67)	1.1 (0.6)
	Last visit	0.53 (0.54)	0.43 (0.45)	0.61 (0.60)	0.73 (0.69)	0.83 (0.51)
HAQ-DI < 0.5, *n* (%)	Baseline	43 (46.7)	21 (51.2)	10 (52.6)	8 (42.1)	2 (40.0)
	Last visit	97 (56.4)	41 (61.2)	23 (53.5)	15 (46.9)	4 (30.8)
HAQ > 1.0, *n* (%)	Baseline	29 (31.5)	12 (29.3)	6 (31.6)	9 (47.4)	3 (60.0)
	Last visit	36 (20.9)	9 (13.4)	11 (25.6)	11 (34.4)	7 (53.8)
Last visit DAS-28 remission, *n* (%)		92 (53.8)	40 (59.7)	20 (48.8)	12 (38.7)	6 (46.2)
Last visit DAPSA-28 remission, *n* (%)		45 (26.3)	17 (25.4)	11 (26.8)	7 (22.6)	2 (15.4)

BASDAI—Bath Ankylosing Spondylitis Disease Activity Index; BASFI—Bath Ankylosing Spondylitis Functional Index DAS-28, Disease Activity Score-28; DAPSA-28, The Disease Activity Index for Psoriatic Arthritis-28; CRP—C-reactive protein (mg/dL); HAQ-DI—Health Assessment Questionnaire Disability Index.

**Table 3:** Distribution of lesions according to vertebral units on lumbar lateral radiograph in PsA.

Location	Osteophyte	Osteophyte ≥ 2	Corner SP	Bridging SP	All SP	Erosion, Sclerosis, Squaring	Ambiguous
T12, L, *n* (%)	7 (3.8)	1 (0.5)	9 (4.9)	5 (2.7)	14 (7.7)	3 (1.6)	2 (1.1)
L1, U, *n* (%)	6 (3.3)	0 (0)	10 (5.5)	5 (2.7)	15 (8.2)	2 (1.1)	2 (1.1)
L1, L, *n* (%)	10 (5.5)	6 (3.3)	8 (4.4)	6 (3.3)	14 (7.7)	4 (2.2)	0 (0)
L2, U, *n* (%)	15 (8.2)	8 (4.4)	8 (4.4)	7 (3.8)	15 (8.2)	4 (2.2)	4 (2.2)
L2, L, *n* (%)	16 (8.8)	6 (3.3)	5 (2.7)	6 (3.3)	11 (6.0)	4 (2.2)	0 (0)
L3, U, *n* (%)	31 (17.0)	13 (7.1)	4 (2.2)	6 (3.3)	10 (5.5)	10 (5.5)	2 (1.1)
L3, L, *n* (%)	17 (9.3)	8 (4.4)	3 (1.6)	5 (2.7)	8 (4.4)	5 (2.7)	2 (1.1)
L4, U, *n* (%)	37 (20.3)	14 (7.7)	8 (4.4)	5 (2.1)	13 (7.1)	11 (6.0)	4 (2.2)
L4, L, *n* (%)	10 (5.5)	1 (0.5)	3 (1.6)	1 (0.5)	4 (2.2)	6 (3.3)	4 (2.2)
L5, U, *n* (%)	24 (13.2)	8 (4.4)	1 (0.5)	1 (0.5)	2 (1.1)	1 (0.5)	1 (0.5)
L5, L, *n* (%)	8 (4.4)	3 (1.6)	1 (0.5)	1 (0.5)	2 (1.1)	0 (0)	0 (0)
S1, U, *n* (%)	3 (1.6)	3 (1.6)	0 (0)	1 (0.5)	1 (0.5)	0 (0)	0 (0)
All vertebral corners (*n* = 2184)	184 (8.4)	75 (3.4)	60 (2.7)	49 (2.2)	109 (4.9)	50 (2.2)	21 (1.0)
All patients	77 (42.3)	33 (18.1)	36 (19.8)	17 (9.3)	44 (24.2)	22 (12.1)	13 (4.7)

SP—syndesmophyte; L—lower; U—upper.

## Data Availability

Data available upon request.
